# “*Without Them, I Would Never Have Been Able to Carry on*” Levers for the Sustained Employment of Patients with Chronic Inflammatory Arthritis: A French Qualitative Study

**DOI:** 10.3390/ijerph192114616

**Published:** 2022-11-07

**Authors:** Chaima Louati, Yosra Mouelhi, Bernard Kabuth, Céline Clément

**Affiliations:** 1 INTERPSY Research Unit, Lorraine University, EA 4432, 54015 Nancy, France; 2 Laboratoire de Santé Publique, Faculté de Médecine, Université Aix-Marseille, EA 3279, 13005 Marseille, France; 3 Centre Psychothérapique de Nancy (CPN), 54521 Laxou, France; 4 CHRU Nancy, Department of Public Health Dentistry, University Hospital, University of Lorraine, 54000 Nancy, France

**Keywords:** musculoskeletal diseases, arthritis, employment, qualitative research

## Abstract

Background. Chronic inflammatory arthritis (IA) is known to be involved in declining work outcomes and increased risk of experiencing unemployment. The aim of this study is to qualitatively identify the levers for the employment of patients with IA and AI-like conditions. Methods. To accomplish this task, a multi-centered, exploratory qualitative design involving one-on-one semi-directed interviews and a focus group was performed among 18 IA French patients to deepen our understanding of what enables patients to maintain employment despite the limiting, chronic, and expanding nature of their symptoms. Results. Analysis revealed five clusters of levers for the employment of chronic IA patients: The first cluster of levers was based on a set of verbatim records mentioning an overall improvement in the management of the disease. The second cluster of levers was based on a set of verbatim records mentioning the perceived added value of one’s occupation at a personal, familial, or societal scale. The third cluster of levers was based on a set of verbatim records mentioning the perceived interpersonal supportiveness of the professional sphere. The fourth cluster of levers was based on a set of verbatim records mentioning the micro-, meso-, and macro-characteristics of the working environment. The fifth cluster of levers was based on a set of verbatim records mentioning intrapersonal attributes. Conclusions. This study deepens and updates the current knowledge on what empowers patients dealing with chronic AI. These results provide valuable insights for stakeholders involved in designing or deploying employment initiatives for patients with AI.

## 1. Introduction

Chronic inflammatory arthritis (IA) conditions were found to have an increased risk of both impairment and unemployment [1]. Through impairing movement, strength, endurance, and fine coordination, these conditions adversely impact productivity and the likelihood of maintaining employment [2]. In addition, the employment impact is potentially noteworthy, since permanent occupational incapacity is prevalent in IA patients [3]. For patients, having such disorders has a major bearing on their overall state of health, their quality of life (QoL), and their ability to work effectively, all of which affect their work attendance, productivity, and whether or not they remain in employment [4]. In addition to the individual repercussions, there are societal ones as well. These are mainly due to the high financial costs resulting from work incapacity, among others reasons. The most recent estimations of the French National Health Insurance Fund, which financially manages the French healthcare system, indicate that the burden of these pathologies reaches an average of EUR 3281 per year and per patient, depending on the type of IA, the method of calculation, and the severity of the functional impairment. This equates to approximately USD 2.6 billion in terms of healthcare expenditures, without counting the missed workdays due to sick leave [5]. Patients reported that despite the limitations, several drivers remain that they can rely on to sustain their work. Some of these facilitators are related to the social value of work [6]. Such patients reported occupational sustainability considerations, which are highlighted in quantitative investigations demonstrating significant associations between long-term unemployment and impaired quality of life [7,8,9]. Therefore, with the Outcome Measures in Rheumatology (OMERACT) initiative, the importance of considering occupational impairment has been emphasized [10]. Thereafter, various epidemiological surveys investigated the predictors of occupational sustainability among such patients. They indicated an association with personal attributes, such as level of education, age, and gender [11]. On the other hand, some studies have linked it to disease-related factors, such as self-reported measures of health conditions [12]. In contrast, fewer studies have more deeply explored subjective patient self-experience. It is thus a priority to understand, from the patient’s perspective, which enablers they perceive as improving their likelihood of sustaining employment [13].

The aim of this survey is to qualitatively investigate the underlying drivers behind the facilitators of employment for patients with IA and other related conditions.

## 2. Materials and Methods

In order to achieve this objective, a prospective and exploratory qualitative study, based on individual semi-directed interviews and focus groups, was carried out with the aim of identifying the levers that promote employment based on the discourse of IA patients [14].

The recommendations of the Consolidated Criteria for Reporting Qualitative Research (COREQ) were followed and applied throughout this study (Supplementary File S1) [15].

**Ethical aspects.** This survey was conducted in conformity with the reference methodology MR004 of the French National Commission for Information Technology and Civil Liberties (CNIL). No further ethics approval was requested by French legislation. Nevertheless, all the participants were provided with an information letter about the study’s aims and data use. Each participant signed a Free Prior and Informed Consent (FPIC) prior to each interview. All data were then pseudonymized, stored, and used in conformance with French laws.

**Sampling.** An effort to maximize the representation of different profiles was made in terms of the type of AI, the length of the illness, the gravity of the symptoms, and the occupational field.

The respondents were recruited from four different French geographical areas, Nancy, Montpellier, Grenoble, and Marseille, using six alternative approaches: (1) local direct recruit by a rheumatologist among her own patients from the Nancy University Hospital (NUH); (2) informational presentation of the project’s objectives and recruitment criteria to the NUH rheumatology staff; (3) outright canvassing of the NUH outpatient medicine unit; (4) diffusion of a call for participation in the newsletter of the French Association for Fight against Arthritis (AFLAR); (5) partnership with the National Association for the Defense of Rheumatoid Arthritis (ANDAR) for focus group recruiting and planning; (6) the use of our network of personal and professional contacts to find appropriate patients in Grenoble and Marseille.

To be eligible for inclusion in our study, the participants had to fulfill our three inclusion criteria: (1) to be aged 18 to 65 years old; (2) to be diagnosed by a rheumatologist as being suffering from rheumatoid arthritis (RA); spondyloarthritis (SpA); or psoriatic arthritis (PsA); and (3) to be employed. Patients who were on sick leave for more than 3 years were not eligible.

### 2.1. Data Collection

A theme-based interview guide was used as the data-collecting method. It was developed by a health psychologist, an occupational psychologist/ergonomist, an occupational physician, and a rheumatologist. Two pilot interviews were carried out to arrive at the final version. The interviewer was trained and supervised by a health psychologist before conducting interviews and focus group sessions. Due to legal requirements, the participants were previously disclosed to the research purpose prior to the interview. The only characteristics shared with the participants about the interviewer were references to her professional qualifications.

In the section of the interview grid that dealt with the facilitators of employment sustainability, the first question was open-ended and generic. It was worded as follows: “What keeps you working despite having your disease?” Subsequently, depending on the interviewee’s answers, the following questions allowed for further exploration of the reported options, such as symptom management, psychological and emotional aspects, the work environment, work settings and conditions, as well as the perceived social support in and outside the workplace. The interviews were conducted using the inductive grounded theory method [16]. The focus group sessions were conducted using the Krueger method [17], which recommends moderating the discussion without orienting answers. They were led by two psychologists: a health psychologist and an occupational psychologist. They alternated between the role of the moderator and the observer. The moderator’s objective was to stimulate the discussion without orienting it. The objective of the observer was to discharge the moderator from various operative tasks that could interfere with his attention, such as note-taking, regularly checking the audio recording, and observing the body language of the participants. All interviews were transcribed verbatim from the audio recordings and pseudonymized before analysis.

### 2.2. Data Analysis

**Figure 1:**
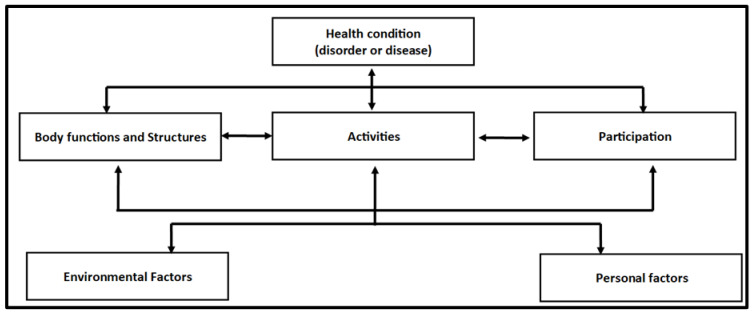
Interaction between ICF–WHO components [19].

Some degree of redundancy started to be noteworthy starting from the 13th interviewee. From that point onwards, data analysis was initiated simultaneously with data gathering. This was undertaken for two methodological considerations: (1) to allow the early tracking of data saturation; (2) to conform with the inductive design as suggested by the Constructivist Critical Grounded Theory (CGT) [18]. The data were analyzed with NVivo^®^ (Version 11, QRS International, Ruggell, Liechtenstein), which is a textual analysis software. The data were then organized through five stages of encoding: (1) unitization of the verbatim records by generating thematic clusters, each one containing a central point that answers our study question; (2) the tagging of each thematic unit with meaningful headings; (3) aggregating the units into prominent categories; (4) ranking the categories according to their centrality in the participants’ statements; (5) identifying underlying dynamics among the thematic categories. The first, second, and third stages of the analysis were carried out separately by two junior researchers. The last two steps were completed jointly by the two junior researchers. The above process was followed by two senior researchers. For better clarity, the themes obtained were then mapped to the five components of the International Classification of Functioning, Disability, and Health (ICF) conceptual scheme [19], as presented in Figure 1.

This classification sets out a standard method to assess the functional state with comparable criteria, a common language, and a standardized framework. It has been approved by all World Health Organization (WHO) member states. It considers that disability is not only a personal problem but also a process of experiencing one’s illness in a given context [20]. The approach states that the overall functioning of a human being in a defined environment is the result of a comprehensive and interactive pattern of health states, social situations, and the surrounding context in which they operate. A dynamic interaction between these entities occurs: Actions in one of these entities potentially affect one or more of the other components. Such interactions are person-specific and do not necessarily fit into a predictable one-to-one pattern. The interactions are rather one-to-many and in two-way directions. It is important to first gather the data on each of these components independently and only afterward are the possible combinations and causal links between them explored. To describe the complete health experience, all components are needed.

## 3. Results

Eighteen people were interviewed through three different modalities.

- Nine participants were interviewed face-to-face in Nancy (France). On-site interviews were made possible by the geographical proximity between the researchers and the participants. This first phase of the survey allowed us to identify the majority of the job retention facilitators.
- Four participants were interviewed through a focus group in Montpellier (France) in partnership with the ANDAR, a patient association. This second step of the survey provided feedback on the first results and allowed us to deepen our observations through discussions and the differences in experiences reported by the participants.
- Five participants were interviewed via phone calls: two in Grenoble (France) and three in Marseille (France). This survey modality was adopted because of the geographical distance between the researchers and the participants. The non-verbal language observation was conceded, as this third phase of the survey was merely to check the accuracy of our observations. It was a way to ensure that we did not miss any important information during the previous phases of the survey. This third phase of the survey also helped to diversify the field and socio-economic contexts.

The study sample is described in Table 1.

The qualitative analysis of the discursive input suggested five major emergent topics of levers to sustained employment for chronic IA patients:

(1) The first cluster of levers was based on the set of verbatim records mentioning an overall improvement in the management of the disease, its related symptoms, and medication observance, all acquired with the advancement of the disease. Through experience, it becomes acquired expertise enabling the proactive management of both symptoms and inflammatory relapses and flare-ups.

(2) The second cluster of levers was based on the set of verbatim records mentioning the perceived added value of one’s occupation at a personal, familial, or societal scale. Such perception can be generated by a high social recognition of one’s profession, by an appealing career or salary, or simply by family necessities.

(3) The third cluster of levers was based on the set of verbatim records mentioning the perceived interpersonal supportiveness of the professional sphere. Such perception is driven by emotional and instrumental coworker support and/or by the procedural and organizational hierarchical supportiveness of the managerial staff and settings.

(4) The fourth cluster of levers was based on the set of verbatim records mentioning the micro-, meso-, and macro-characteristics of the working environment:

- At the micro-individual scale: ergonomic workstation design or flexible scheduling;
- At the meso-professional level: inclusiveness policies implemented by one’s organization, awareness and acquaintance of one’s coworkers regarding invisible disabilities, the presence of other coworkers with disabilities or similar illnesses, and in the present case, the pathologies affecting the locomotor apparatus;
- At the macro-societal scale: national legislation promoting employment retention among vulnerable workers, fiscal and other financial incentives to accommodate or reclassify such workers, and coercive sanctions against unfair or unjustified dismissals of such workers.

(5) The fifth cluster of levers was based on the set of verbatim records mentioning intrapersonal attributes, such as the following features:

- The perceived self-efficacy owing to post-diagnosis occupational achievements;
- A resilient and assertive temperament;
- Having experienced other well-managed or successfully treated comorbidities.

Every emergent topic, its underlying grounds, and the illustrating verbatim records are detailed in Table 2, and the correspondence of the emergent topics from data analysis with the ICF’s components is described in Table 3.

The emergent item regarding the private sphere or social sphere beyond the occupational context was not considered since it was outside the scope of the study, which was exclusively focused on the occupational repercussions of the disease. This item would fit the “Activities” component of the ICF–WHO referring to the ability to accomplish basic tasks or actions of everyday life.

**Figure 2:**
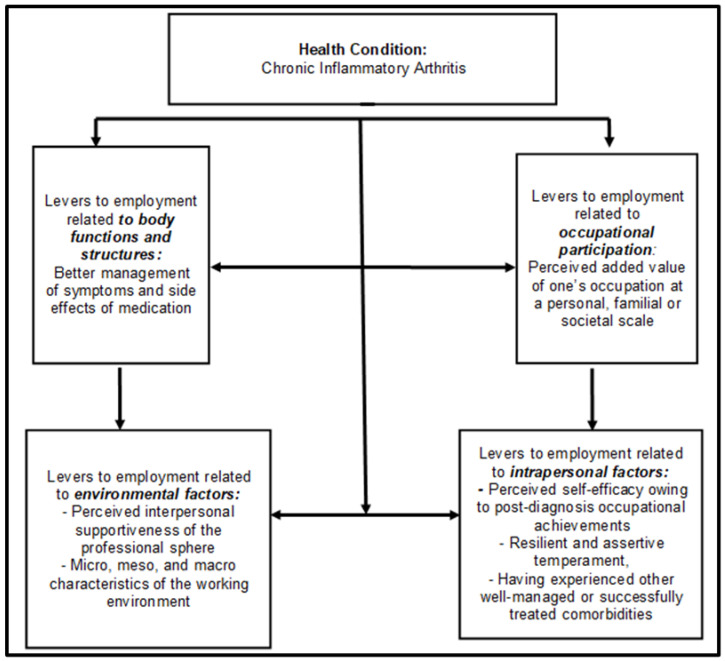
Levers for employment of patients with IA structured in accordance with the ICF–WHO model.

In terms of matching the emergent themes of the data analysis to the ICF–WHO model, the obtained pattern is presented in Figure 2.

## 4. Discussion

Our observations are consistent with those of Purc-Stephenson et al. [21], who conducted a systematic review of 17 qualitative studies involving 873 workers with chronic inflammatory arthritis. Their study revealed 11 themes reported by employees with IA. These themes were then clustered into four categories: (1) the strategies adopted to cope with the changing nature of the disease and the unpredictability of symptoms; (2) the strategies adopted to improve one’s perceived self-efficacy; (3) the strategies adopted to improve one’s ability to communicate about one’s illness to coworkers; and (4) the strategies adopted to enhance one’s organizational maturity toward persons with impairments.

From a closer point of view, the perceived social support component appears to be unavoidable, as it was mentioned by all the participants in our survey. Indeed, whether or not perceived social support exists, it appears to be a decisive factor in the job retention of patients with IA. This observation resonates with the very basis of the “job demand–control–support” model of Karasek et al. [22]. This model places the role of social support from the occupational sphere at the center of the interaction between the job requirements and the ability of an individual to fulfill them. As perceived social support increases, an individual’s ability to cope with his job requirements increases accordingly [22]. In the same sense, the centrality of perceived social support is more widely acknowledged in a meta-analysis conducted by Stergiou-Kita et al. [23]. Through a systematic assessment of 39 studies investigating determinants of job retention in cancer survivors, it was revealed that hierarchical recognition and peer support significantly affect the patient’s perceived valuation of occupational recovery.

Furthermore, the consideration of the patient’s experience is highlighted in the latest recommendations of the European Alliance of Associations for Rheumatology (EULAR) for the implementation of self-management strategies in patients with inflammatory arthritis. More specifically, this awareness of the patient’s voice is reflected in the seventh recommendation, which calls on healthcare professionals to involve patients in work-related debates and refer them to supportive resources. Involving them in the proceedings is an important way to help them maintain their employment and thus their independence. EULAR also encourages the participation of ergonomists and occupational health experts in the discussions to provide targeted guidance and practical information to assist in the diagnosis and treatment of the disease. Effectively, the target of EULAR up until 2023 is to enhance the work participation (WP) of persons with rheumatic diseases [24].

In addition to the benefits to the patients, supporting job retention also benefits employers by reducing costs resulting from presenteeism or absenteeism. Indeed, keeping experienced employees is more cost-effective than replacing them with newcomers who still need to be technically trained and organizationally integrated [25]. In a win–win approach, a better level of awareness will allow employers to act earlier to minimize eventual complications leading to unproductive attendance or extended absences. The employer has everything to gain by taking a supportive stand.

However, it should be noted that the impact of perceived social support on job retention is less significant among young adults with AI. In this regard, a study conducted by Jetha et al. [26] showed that young adults with IA attribute lower value to social support, career stability, and long-term job-keeping. Essentially, they have the same attitudes regarding work–life as their non-diseased peers. The lever of *“perceived social support”* seems then less relevant to promote the employment of young adults with AI.

In terms of limits, we acknowledge that despite attempts to maximize inclusiveness, our sampling was female-dominated; thus, the male perception was not accurately represented. Retrospectively, we consider that the male under-representation represents an effective deficit of information, especially since a recent study by Pinheiro et al. [27] revealed that regardless of similar symptomatology, gender influences the diagnosis, treatment, and activity measures of the disease. In this case, it is about spondyloarthritis. We further acknowledge a perimeter leak in the process of broadening our recruitment. This is because patients with early IA who missed the diagnostic criteria due to seronegative IA or mono-arthritic symptoms were not included. We further concede that most participants operated in a middle-class environment, which implies that we may have missed other challenges typically faced by less skilled categories such as unskilled workers, farmers, or laborers. These same participants work mostly in urban areas, which excludes the actual challenges faced in rural areas. Meanwhile, a longitudinal follow-up of over 10 years demonstrated that low income or rural residence has the potential to be a risk factor for an increased prevalence of the most common form of IA, rheumatoid arthritis, versus high socio-economic status or urban residence [28].

## 5. Conclusions

The authors believe that these results can be useful in two complementary ways. The first is that they can be used to assess a patient’s professional stability. The more facilitators the patient accumulates, the better his or her occupational stability would be. The fewer facilitators the patient accumulates, the more vigilant the occupational health professionals who supervise him or her should be. The other way of exploiting our results is to use the identified facilitators as levers of action to be activated in order to design personalized job maintenance interventions.

## Figures and Tables

**Table 1:** Sample description.

Type of IA				Socio-Professional Category (SPC)			Centers (France)	Sex Proportion (Women/Men)	Mean Age (Years)
PR ****	JIA ***	RA **	SpA *	High SPC	Medium SPC	Low SPC			
0	1	3	5	2	6	1	9 Nancy	7/2	43.4
3	0	1	0	1	2	1	4 Montpellier	1/3	46.2
0	0	1	1	0	1	1	2 Grenoble	1/1	45.0
0	0	1	2	0	0	3	3 Marseille	2/1	43.3
**3**	**1**	**6**	**8**	**3**	**9**	**6**	**18**	**11/7**	**44.2**

SPCs are ranked in accordance with the official French nomenclature of professional fields and socio-professional categories. * SpA: spondyloarthritis. ** RA: rheumatoid arthritis. *** JIA: juvenile idiopathic arthritis. **** PR: psoriatic rheumatism.

**Table 2:** Emergent topics of the analysis.

Emergent Topics	Underlying Grounds	Illustrating Verbatim Records
**1. Levers related to improvement in disease management**	- Better symptom management and medication observance, acquired with the advancement of the disease	- *“With time, I know almost as much [about the disease] as my rheumatologist.” (Woman, 36 years old, Jurist, Nancy)* - *“I now know what causes me to have flare-ups and when, and I make arrangements accordingly.” (Woman, 48 years old, Nurse, Nancy)* - *“On the IV injection days, I don’t plan to do anything major. I already know that I will be completely out of action.” (Woman, 38 years old, speech-language pathologist, Nancy)*
**2. Levers related to the perceived added value of one’s occupation at a personal, familial, or societal scale.**	- High social recognition of one’s profession, by an appealing career or salary, or simply by family necessities.	- *“I can’t put my team in trouble. They rely on me.” (Woman, 46 years old, nurse, Nancy)* - *“I do love my students and they give it back to me. That’s what keeps me going.” (Man, 43 years old, Teacher, Nancy)* - *At the end of my day [of work] I feel like I’m doing something useful. At least* *I’ve accomplished something. I can’t do without that feeling [of accomplishment]. This is why I chose to do this job [nursing]. That’s why I’m still doing it anyway.” (Woman, 48 years old, Nurse, Nancy)* - *“Who is going to pay my bills if I give in [to illness]? When you have kids to take care of, you can’t think only of yourself. “ (Man, 56 years old, Baker, Marseille)*
**3. Levers related to the perceived interpersonal supportiveness of the professional sphere**	- Emotional and instrumental coworker support; - Procedural and the organizational hierarchical supportiveness of the managerial staff and settings.	- *“Without them [colleagues] I would never have been able to carry on [working].” (Man, 34 years old, factory worker, Grenoble)* - *“I no longer have to carry any heavy items. Every time someone spontaneously offers to carry them for me before I even ask.” (Woman, 46 years old, factory manager, Marseille)* - *“I made an appointment, talked to my supervisor. I felt better just for having been heard. Better, before things were put in place. Since then, we’ve been checking in regularly, and it’s comforting to know that we’re being heard.” (Woman, 48 years old, Nurse, Nancy)*
**4. Levers related to the characteristics of the working environment**	- At the micro-individual scale: ergonomic workstation design or flexible scheduling; - At the meso-professional level: inclusiveness policies implemented by one’s organization, awareness and acquaintance of one’s coworkers regarding invisible disabilities, the presence of other coworkers with disabilities or similar illnesses, and in the present case, the pathologies affecting the locomotor apparatus; - At the macro-societal scale: national legislation promoting employment retention among vulnerable workers, fiscal and other financial incentives to accommodate or reclassify such workers, and coercive sanctions against unfair or unjustified dismissals of such workers.	- *“I saw that it [the implementation of facilities] took time. I bought the most urgent ergonomic tools myself.”(Woman, 36 years old, Jurist, Nancy)* - *“When I noticed how they [management] treat colleagues, it made me feel more comfortable. It encouraged me to talk about it [his illness].” (Man, 34 years old, factory worker, Grenoble)* - *“I am the very first to criticize the [French] system. It’s true that there are still things to improve, but I admit that I feel quite privileged because if I were in another country I wouldn’t be as well supported.” (Man, 56 years old, administrator, Montpelier)*
**5. Levers related to intrapersonal** **attributes**	- Perceived self-efficacy owing to post-diagnosis occupational achievements; - A resilient and assertive temperament; - Having experienced other well-managed or successfully treated comorbidities.	- *“I am hoping that science will be able to provide a remedy for my condition. I have faith. A treatment will ultimately be found. I keep up with the latest developments and share them with other patients. It helps keeping my mood up.” (Woman, 38 years old, speech-language pathologist, Nancy)* - *“You can’t let it get you down. You have to go on living. I have to accept it, I have to live with it and I have to go on living.” (Man, 36 years old, self-employed, Nancy)* - *“I have experienced others [illnesses] you know and I have always managed to take control of my life. I pay attention to my lifestyle. It does make a difference.” (Man, 56 years old, Baker, Marseille)*

**Table 3:** Correspondence of the emergent topics from data analysis with ICF components.

ICF Components’ Definition	Correspondence with ICF Components	Emergent Topics from Analysis
It refers to impairments and problems with body functions or anatomical systems, manifested by a significant gap or loss.	**Body functions and structures**	1. Levers related to the improvement in the management of the disease, its related symptoms, and medication observance
It refers to the ability to take part in a social, familial, or professional real-life situation.	**Participation**	2. Levers related to the perceived added value of one’s occupation at a personal, familial, or societal scale
It refers to the physical, social, and attitudinal environments in which people live and conduct their lives.	**Environmental** **Factors**	3. Levers related to the perceived interpersonal supportiveness of the professional sphere
		4. Levers related to the micro-, meso-, and macro-characteristics of the working environment
refers to the specific living environment of an individual, consisting of characteristics of the person that are not part of a health problem or condition	**Personal factors**	5. Levers related to intrapersonal attributes

## Data Availability

Raw data and data collection tools are fully available upon request. Chaima Louati should be contacted if someone wants to request the data from this study.

## Supplementary Materials

The following supporting information can be downloaded at: https://www.mdpi.com/article/10.3390/ijerph192114616/s1, File S1: COREQ (COnsolidated criteria for REporting Qualitative research) Checklist.
